# Defect Filling Method of Sensor Encapsulation Based on Micro-Nano Composite Structure with Parylene Coating

**DOI:** 10.3390/s21041107

**Published:** 2021-02-05

**Authors:** Jialin Yao, Wenjiang Qiang, Xingqi Guo, Hanshui Fan, Yushuang Zheng, Yan Xu, Xing Yang

**Affiliations:** 1 The State Key Laboratory of Precision Measurement Technology and Instruments, Department of Precision Instrument, Tsinghua University, Beijing 100084, China; colin-yao@foxmail.com (J.Y.); guoxq20@tsinghua.org.cn (X.G.); 2 School of Materials Science and Engineering, University of Science and Technology Beijing, Beijing 100083, China; fanhanshui@sjtu.edu.cn (H.F.); 41703271@xs.ustb.edu.cn (Y.Z.); 41703059@xs.ustb.edu.cn (Y.X.)

**Keywords:** defect filling method, composite structure, parylene coatings, MEMS, pressure sensors

## Abstract

The demand for waterproofing of polymer (parylene) coating encapsulation has increased in a wide variety of applications, especially in the waterproof protection of electronic devices. However, parylene coatings often produce pinholes and cracks, which will reduce the waterproof effect as a protective barrier. This characteristic has a more significant influence on sensors and actuators with movable parts. Thus, a defect filling method of micro-nano composite structure is proposed to improve the waterproof ability of parylene coatings. The defect filling method is composed of a nano layer of Al_2_O_3_ molecules and a micro layer of parylene polymer. Based on the diffusion mechanism of water molecules in the polymer membrane, defects on the surface of polymer encapsulation will be filled and decomposed into smaller areas by Al_2_O_3_ nanoparticles to delay or hinder the penetration of water molecules. Accordingly, the dense Al_2_O_3_ nanoparticles are utilized to fill and repair the surface of the organic polymer by low-rate atomic layer deposition. This paper takes the pressure sensor as an example to carry out the corresponding research. Experimental results show that the proposed method is very effective and the encapsulated sensors work properly in a saline solution after a period of time equivalent to 153.9 days in body temperature, maintaining their accuracy and precision of 2 mmHg. Moreover, the sensors could improve accuracy by about 43% after the proposed encapsulation. Therefore, the water molecule anti-permeability encapsulation would have broad application prospects in micro/nano-device protection.

## 1. Introduction

Micro/nano film coating has many important applications in device encapsulation and protection [1,2,3,4,5]. The coating has the advantages of light structure, good corrosion resistance, high adhesion, and good reliability, which can improve surface performance and increase the service life of the device [6,7]. Many devices work in special or harsh environments, such as water or a humid environment, where the effects of liquids on the encapsulated coating and devices must be considered [8,9]. Therefore, studying how to use the micro/nano film coating for waterproof encapsulation protection to ensure the normal operation of the devices for a long time has great value [10,11,12].

At present, micro/nano film coatings are generally made of micron-level thickness polymer materials [13,14,15]. Parylene C is favored as a representative of this material. Active molecules with good penetration can form air-gap-free micron layers within, at the bottom, and on the surface of devices because Parylene C coatings are often prepared by chemical vapor deposition. Parylene C can be deposited at room temperature and has low permeability to moisture, good corrosion resistance, and biological compatibility. The film coating does not substantially increase the external size of devices; thus, Parylene C has become a common polymer material in device protection materials. As an example in the field of wearable electronic devices, Parylene C coatings can protect their precision components and will not affect the function of circuit board components, enabling the waterproof performance of devices to achieve Ingress Protection X8 (IPX8) [16], which means the test time is at least one hour when immersed at least one meter below the surface of the water. It can even resist acid, alkali, and organic solvent, and has excellent barrier and protection ability to water vapor, salt spray, and other harsh environments. In conclusion, a very thin film coating of Parylene C provides good protection to ensure the monitoring accuracy and service life of wearable electronic devices. Parylene C film coatings can be used for medical device protection. Medical devices coated with Parylene C can possess improved surface lubricity and biocompatibility to ensure their reliability in a damp environment [17,18]. In the body fluid environment, Parylene C film coatings can prevent the circuit in the devices from being damaged by water vapor to ensure that it can work stably in the salt solution environment implanted in the body and has good biological compatibility to ensure implantation safety [19].

However, Parylene C film coatings need to be improved in terms of duration and effectiveness of long-term waterproofing mainly because Parylene C is a kind of polymer material, and its arrangement of molecular chains is not as dense as the atoms of metal and ceramic materials, and material preparation defects are unavoidable in production. Thus, Parylene C film coatings still possess certain water permeability, which is very unfavorable for long-term waterproof protection devices [20].

A study in 2020 by Li and Cauwe et al. [21] prepared and analyzed a highly moisture-proof Polyimide (PI)/(HfO_2_/Al_2_O_3_/HfO_2_)(ALD-3)/PI structure packaging material. Al_2_O_3_ and HfO_2_ layers were deposited by an atomic layer deposition (ALD) process at 250 °C, in the form of an ALD-3 stack. The copper protected by ALD Al_2_O_3_ and ALD-3 is effective at temperatures below 70 °C, and a life span of 37 °C can be estimated. After soaking in Phosphate Buffered Saline (PBS) at 60 °C for more than 1215 days, the PI/ALD-3/PI barrier did not degrade. Xie and Rieth et al. evaluated the long-term reliability of a dual-layer coating package with 52 nm ALD Al_2_O_3_ and 6 μm Parylene C deposited sequentially for the neural interface of the Utah electrode array (UEA), and compared it with the use of only parylene coating. The packaged devices were compared. The accelerated life test was carried out in PBS at 57 °C. The equivalent immersion time of double-layer coated devices at 37 °C was as long as 1044 days, while the package using only Parylene C had only about 100 days of life. Moisture diffusion and interface pollution are important reasons for Parylene C packaging failure [22,23]. However, those studies are mainly aimed at the encapsulation method of the non-movable part of devices, while the encapsulation method and internal mechanism of the movable part of devices have seldom been studied in depth. For example, a pressure sensor with the movable pressure-sensitive membrane will be repeatedly deformed under the action of a cyclic load when it is working, and the encapsulation coating will affect the movement and work of the movable pressure-sensitive membrane, causing the sensor output to drift.

In the actual working process, liquid gradually reaches the surface of devices through permeation in Parylene C thin film, causing device failure. Thus, analyzing the penetration mechanism of liquid in the Parylene C coating is important, and allows us to improve the effectiveness of water molecule anti-penetration. In simple terms, water molecules in polymer materials can pass through the instantaneous molecular chain gap caused by the thermal movement of the molecular chain, diffusing in the free volume of the chain and slowly accumulating on the surface of the devices after a long infiltration time. For devices with waterproof requirements, oxidation and corrosion of several materials in the devices will occur when the moisture content on the surface of the devices exceeds a certain value, eventually leading to the destruction and failure of the device. Encapsulation coating materials may also cause interface stratification, aging cracking, and other problems with the penetration of water molecules, thereby accelerating water penetration. Avoiding the surface and internal defects of the materials prepared is also difficult, and the high surface energy at the defects accelerates the diffusion of water molecules along with the defects [24,25].

According to this problem, this paper studies and analyzes the water penetration mechanism of Parylene C coatings. On this basis, we put forward a kind of defect filling method of micro-nano composite structure and encapsulation method for the water permeability of Parylene C. Surface defects of Parylene C coating are covered and filled by depositing nanoscale oxide coating on Parylene C thin film to improve the waterproof permeability of the encapsulation coating effectively [26]. A controlled experiment was designed to verify the waterproof capability of the method by taking an implantable pressure sensor as an example. Waterproof times of medical pressure sensors under three thin-film coating encapsulation methods, namely, the nano-Al_2_O_3_ layer, the micron polymer Parylene C layer, and the defect filling method of the micro-nano composite structure layer, were compared. The experimental results show that the waterproof performance of the device was improved substantially by using the micro-nano composite structure layer. Furthermore, the influence of the packed micro–nano composite encapsulation layer on the error and sensitivity were studied, as well as the variation of the error in the simulated body fluid. The results provide a reference for the current encapsulation protection and improvement of medical devices [27].

## 2. Permeation Mechanism

### 2.1. Analysis of the Permeation Mechanism in Parylene C Encapsulation Coating

Parylene C is a linear, unbranched, and highly crystalline polymer material with good water vapor barrier properties. It has become a commonly used moisture-proof protective coating in implantable device encapsulation. The permeation mechanism of Parylene C coating was analyzed by using the diffusion principle and free volume theory [28].

From a microscopic point of view, the permeation of water molecules in Parylene C is essentially a diffusion movement of molecules. Diffusion is a migration caused by the irregular thermal motion of molecules. In a humid environment, the diffusion of water molecules in polymer Parylene C is driven by the concentration gradient of water molecules as diffusion agents; thus, this diffusion belongs to transfer diffusion under a non-equilibrium system [29].

Researchers have proposed various models such as the free volume model, barrier model, and concentration-dependent model to study this diffusion behavior better [30]. The permeation of water molecules in Parylene C is carried out through the voids in the polymer. The size and distribution of the free volume of the polymer play a key role in the diffusion of water molecules in the polymer. Therefore, we use the free volume theory for analysis.

The free volume model identifies that the interior of polymer materials can be considered to consist of three parts: the volume occupied by polymer chains, the free volume existing between polymer chains, and the gap volume generated by the chain movement. Free volume is defined as the ratio of blank volume in the membrane to total volume and is related to external parameters such as temperature and pressure. According to relevant research, the diffusion of water molecules in polymer films is not continuous motion but continuous reciprocating motion over a small distance and jumping motion over a large distance, which conforms to the hole jump-diffusion theory; that is, diffusion is not continuous. In this process, the size and distribution of free volume inside the polymer film greatly influence the diffusion rate of small molecules in the film. The mechanism of jump diffusion involves the polymer segments around the permeated molecules. Although simulation calculation generally considers that a “pore” structure will be formed between two consecutive penetration positions in the polymer, the “pore” structure may not be strictly formed, but the permeated molecules and their surrounding polymer segments share a certain volume before and after the diffusion jump. The diffusion behavior of small molecules in the film has two forms: the tiny movement of small molecules in the free volume voids between polymer chains most of the time and the jump diffusion of small molecules between the free volume voids in the polymer. First, small molecules vibrate in the free volume voids. When the thermal motion of the flexible polymer chain forms a temporary channel connecting the two free volume voids, the water molecules that have gained energy may jump to another small space to complete the jump diffusion [31,32,33,34]. Although individual molecules can diffuse in any direction, their motion is Brownian motion, but small molecules will spontaneously undergo entropy increase under the influence of driving force (such as the concentration gradient in this experiment), resulting in macroscopic inward diffusion [33,34,35].

The results of simulation calculations show that free volume voids with a radius greater than 1 Å in Parylene C are less than 10% of the total volume, while the voids with a radius greater than 2 Å account for only about 1.5% of the total volume [36]. Theoretically, Parylene C polymer has good barrier properties to water molecules because the diffusion of small molecules in polymer voids is closely related to their van der Waals radius, and the van der Waals radius of H_2_O molecules is around 3 Å. Parylene C is relatively dense in polymer materials and the composition of molecular chains does not contain hydrophilic groups, which makes the material itself highly hydrophobic. Therefore, the entire Parylene C film ideally reflects excellent water vapor barrier performance.

However, the Parylene C film material actually fabricated cannot be equivalent to the ideal model, and the permeation of water molecules in Parylene C encapsulation coating is influenced by other factors. For example, in actual production and use, surface defects such as microcracks and pinholes usually occur on the surface of materials due to preparation, environmental conditions, structural composition, preparation thickness, and other reasons. Parylene C has good insulation properties [11,37], which also makes it difficult to observe the cracks and defects on the surface under the electron microscope. Previous studies showed that Parylene C films with a thickness of less than 5 µm may have defect-driven permeation and the degree of permeation of water molecules through defects may be greater than the general overall diffusion level [38], motivating us to focus on the influence of preparation defects of Parylene C on water permeation. Surface defects will facilitate the adsorption of water molecules and accelerate the condensation of water molecules at defects. Moreover, the energy at the surface defect is higher than that inside the material, which will provide a greater diffusion driving force to make water molecules diffuse faster along with the defect, helping small molecules quickly enter the material. Defects inside the material also accelerate the condensation and diffusion of water molecules inside the material. Therefore, Parylene C film coating needs to be further improved in terms of long-term waterproof time and effect.

In summary, if the free volume of the material can be artificially reduced, the effective area where small molecules can jump and diffuse can be reduced, the number of defects on the surface and inside can be reduced, the crystallinity of the polymer material can be improved, and water molecules will have greater difficulty diffusing between polymer chains. This method can improve the impermeability of the prepared Parylene C material. However, considering that adjusting the free volume of the prepared Parylene C is not easy, we propose a defect filling method of micro-nano composite structure to solve surface defects and improve the waterproof ability of the encapsulation.

### 2.2. Defect Filling Method of Micro-Nano Composite Structure

**Figure 1:**
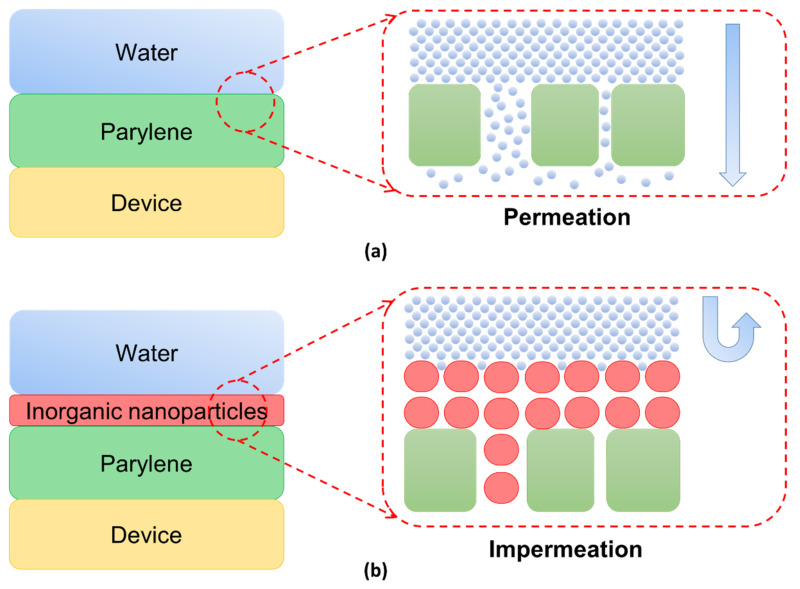
(**a**) Schematic diagram of water permeation of parylene encapsulation layer; (**b**) schematic diagram of waterproof permeation principle of defect filling method of micro-nano composite structure layer.

Analysis based on the permeation mechanism of Parylene C coating shows that the permeation rate of water molecules in the polymer film is affected by the size of the free volume inside, and the number of surfaces and internal defects. We propose a double-layer encapsulation method with a filling mechanism for Parylene C defects because the deposited Parylene C has preparation defects such as pinholes and micro-cracks on the surface of the material. The deposition schematic diagram of the encapsulation method is shown in Figure 1b, where the structure of the defect filling method is realized by depositing dense inorganic oxide nanoparticles layer on the Parylene C film by a low deposition rate method. In Figure 1a, the original preparation defects such as pinholes and micro-cracks on the surface of Parylene C will accelerate the permeation of water molecules. While these original preparation defects will be filled effectively by inorganic oxide nanoparticles in this encapsulation method shown in Figure 1b.

From the perspective of the anti-permeability mechanism of the method, on the one hand, the overall waterproof ability of the encapsulation increases due to the addition of the dense nano-scale inorganic oxide layer. On the other hand, the surface defects of Parylene C polymer film will be filled by nano-scale inorganic oxide particles at a low deposition rate. This makes the microscopic defects fill with nanoparticles and decompose into many smaller voids, which make the permeation space smaller, thus effectively hindering the rapid permeation of water molecules through the defects.

After a nano-scale inorganic oxide layer is applied to the surface of Parylene C, according to the proposed mechanism, we found that most defects such as pinholes and microcracks on and near the surface of Parylene C were filled with inorganic oxide nanoparticles, thus repairing Parylene C to a certain extent. Moreover, the interfacial gaps between the two films were reduced due to the filling of the defects to reduce the probability of water molecules nucleating and accelerating condensation in the interfacial gaps. The interface had less room for water molecules; thus, it can effectively delay the permeation of water molecules and further affect the rate of the entire permeation.

## 3. Materials and Methods

### 3.1. Selection of Encapsulated Devices

We obtained the encapsulation method based on the penetration mechanism of the Parylene C encapsulation method and the defect filling method of micro-nano composite structure, then we designed a series of verification tests. The reliability of the implantable medical devices must be considered to avoid the danger of working in the human environment. Therefore, in this experiment, we used such devices as an example to verify the proposed method. Among medical implantable devices, a large class of implantable devices has a movable structure, such as a mechanical quantity sensor and actuator. This large class of implantable devices has higher requirements for encapsulation technology due to the movable structure. Compared with ordinary devices, the waterproof encapsulation of implanted devices usually needs to meet three basic conditions: (1) implantation safety, (2) waterproof ability, and (3) work performance after encapsulation. Especially, higher requirements are proposed for the waterproof ability of the encapsulation layer of the implanted devices with movable parts, where the encapsulation cannot have a great influence on the motion of the movable structure in the device to ensure the working performance, that is, the encapsulation layer cannot be very thick. The encapsulation material must be flexible to ensure that the sensitivity and accuracy of the sensor will not be excessively lost.

A kind of implantable pressure sensor with movable parts was selected as the experimental device.

This kind of implantable pressure sensor can be implanted into the body to continuously monitor the body’s pressure for a long time. In actual clinical use, the service life of the device in the human body needs to reach months or even years. Therefore, compliant encapsulation is essential for the long-term stable operation of implantable pressure sensors in vivo.

### 3.2. Selection of Encapsulation Materials

Parylene is a commonly used polymer encapsulation material. As mentioned above, the waterproof encapsulation of the implantable pressure sensor also needs to meet at least three conditions: (1) implantation safety, (2) waterproof ability, and (3) work performance, and the two latter requirements need a higher guarantee than in ordinary devices. Parylene is a type of semi-crystalline hydrophobic polymer and often used for encapsulation and protection due to its properties (such as biocompatibility, hydrophobicity, body fluid stability, and good mechanical properties). Referring to Table 1, we selected Parylene C film as the qualified encapsulation.

Parylene can deposit conformal films using chemical vapor deposition (CVD). Commonly used types of parylene include Parylene C, Parylene D, Parylene N, and Parylene HT. Parylene C has a chlorine atom on the benzene ring, which is chemically inert and has low moisture permeability. Although its temperature resistance is lower in several parylenes, Parylene C is a Class VI material in the United States Pharmacopoeia and has the highest biocompatibility certification of plastic materials. Parylene C also has good micromachining compatibility and is commonly used in Micro-electromechanical Systems (MEMS) devices. As a flexible polymer material, it also exhibits mechanical flexibility and low intrinsic stress, coupled with the use of vacuum vapor deposition to prepare higher-quality Parylene C film. In general, Parylene C film can protect the movable structure of the sensor, and it ensures that the sensitivity and accuracy of the sensor will not be excessively lost. In summary, the selection of Parylene C is more consistent with the encapsulation requirements for implantable pressure sensors in this experiment.

We selected dense alumina (Al_2_O_3_) material with good waterproof ability as the nano-scale inorganic oxide layer in the defect filling method. For the low-rate deposition method indicated in the structure, we selected the atomic layer deposition (ALD) technique. The nano-scale Al_2_O_3_ film prepared by atomic layer deposition technology not only has a dense structure, few defects, and excellent waterproof penetration performance but also can cover irregular surfaces highly conformably with high film quality. The dense Al_2_O_3_ film has low mechanical flexibility, which may cause adverse effects on the sensitivity and accuracy of pressure sensors with movable parts. However, in the above defect filling method, Parylene C flexible material is used as the bottom buffer layer to separate Al_2_O_3_ from the sensitive element, which can also reduce the loss of pressure sensor sensitivity and accuracy. Next, we verified it through experiments.

### 3.3. Encapsulation Process Method

To verify the proposed method and achieve new encapsulation methods, we designed a comparative experiment and fabricated three groups of devices in this paper: (1) group of only Parylene C encapsulation: 1 um Parylene C; (2) group of only alumina encapsulation: 50 nm Al_2_O_3_; and (3) group of defect filling method of micro-nano composite structure encapsulation: 1 um Parylene + 50 nm Al_2_O_3_. The fabricated device was placed in the physiological saline solution, and working life time and change of error were recorded regularly.

All reagents and solvents are commonly available materials and can be used without further purification. The SM5420 sensor of SMI Company was used as an example in the experiment. The encapsulation material was Parylene C, Al_2_O_3_, and Epoxy AB adhesive (the main component is the epoxy resin). The specific process was as follows:

(1) Parylene C encapsulation. The special coating system (Specialty Coating Systems, Inc.) PDS2010 was selected to form Parylene C coating film with strong adhesion on the surface of SM5420 pressure sensor using the following steps: First, the SM5420 pressure sensor was placed in the vacuum deposition chamber of the Parylene special coating system to form a nano-level A-174 coupling agent molecular layer on the surface of the device. Next, the Parylene C raw material was placed in the evaporation chamber of the Parylene special coating system. When the vacuum degree in the vacuum deposition chamber of the Parylene special coating system reached 10 mTor, the raw Parylene C material in the evaporation chamber was heated. When the temperature reached to 175 °C, Parylene C raw material was sublimated. The gaseous Parylene C obtained by sublimation entered the cracking chamber of the Parylene special coating system. When the temperature of the cracking chamber reached 690 °C, the gaseous Parylene C were cracked into gaseous monomers. The gaseous monomer entered the vacuum deposition chamber, deposited, and polymerized on the surface of the pressure sensor to form a Parylene C polymer coating.

(2) Al_2_O_3_ encapsulation. The device was placed in an atomic layer deposition chamber, and the deposition material was deposited layer-by-layer in the form of a monoatomic film on the periphery of the device at a deposition temperature of 200 °C. We used ALD deposition equipment (Sentech SI-ALD) for the plasma deposition process, the environment temperature was 200 °C, and the deposition time was 1.5 h. After completing the process, we achieved the deposition of 50 ± 1.5 nm Al_2_O_3_. The coating thickness was measured using an in-situ ellipsometer.

(3) Defect filling method of micro-nano composite structure encapsulation. Al_2_O_3_ atomic layer deposition was applied to the device after coating with Parylene C.

In the experiment, the thickness of Parylene C coating was measured by a Dektak XT step instrument, its working principle is to use a probe to measure the thickness of the film. When measuring, the Parylene C film was placed on the step instrument to make the probe pass near the edge of the step. The probe fluctuated when passed through the junction. The circuit was used by converting the fluctuations into electrical signals and the thickness of Parylene C film could be measured. After setting the chamber pressure and power, several points were randomly selected, and the average value of the coating thickness was 1 μm. At the same time, the experiment used an ellipsometer to measure the thickness of nano-scale Al_2_O_3_. Because the ellipsometer was not in contact with the sample during the measurement, there was no damage to the sample. Thus, this method is widely used in the thickness measurement of nano-scale materials. After the measurement, the average thickness of the Al_2_O_3_ coating was 50 nm.

### 3.4. Test of Waterproof Time

To improve the experimental efficiency, this paper adopted accelerated waterproof test experiments by higher temperatures. In the experiment, 67 °C was selected as the accelerated aging temperature, and its aging rate was eight times that of 37 °C body temperature. The sensor encapsulated with Parylene C was soaked in normal saline and device error was tested regularly. Moreover, we defined the sensor error as being beyond 2 mmHg as the pressure sensor damage standard.

**Figure 2:**
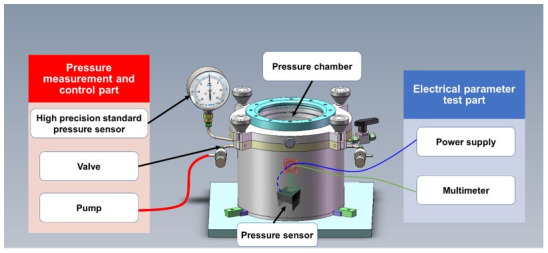
Diagram of the pressure sensor measurement system.

A pressure sensor measurement system was designed to test the error of the pressure sensor, as shown in Figure 2. The pressure sensor measurement system was mainly composed of pressure measurement, control part, pressure chamber, and electrical parameter measurement part, which can record the data of the pressure sensor under the given pressure. The system can control the gas in and out through the opening of the pneumatic valve in the pressure control device. The accuracy index (including nonlinearity, repeatability, hysteresis, and other parameters) of the pressure sensor were obtained by measuring the output voltage of the sensor chip under different pressure values with a periodic change in pressure. Then, the measurement error of the pressure sensor was calculated according to the accuracy index obtained from experiments.

The error testing method is as follows: in a room temperature environment, the sensors which need to be measured were placed in the pressurized cabin. No fewer than three circulation preloads were applied to the sensors, then six measurement points were selected at equal intervals within the full range including the upper and lower process of the sensor measurement, and the output corresponding to the input pressure point was recorded. The pressure measurement range was 0 to 75 mmHg and the pressure interval was 15 mmHg. The sensor output corresponding to the input pressure point was recorded, and the cycle was repeated more than four times. The least-square method was used to obtain the relationship curve between the sensor output voltage and the pressure, where the slope is the sensitivity. The error value is related to three error sources derived from non-linear error (*NL*), hysteresis (*H*), and repeatability (*R*). Total error value (*E_T_*) is obtained by the sum of the squares of the three types of error sources [40]:

(1) $$E_{T}=\sqrt{{\left(NL\right)}^{2}+{\left(H\right)}^{2}+{\left(R\right)}^{2}}$$

After processing the test results, the errors of the pressure sensor before and after encapsulation were obtained.

## 4. Results

### 4.1. Comparison of Waterproofing Time of Different Encapsulation Methods

**Figure 3:**
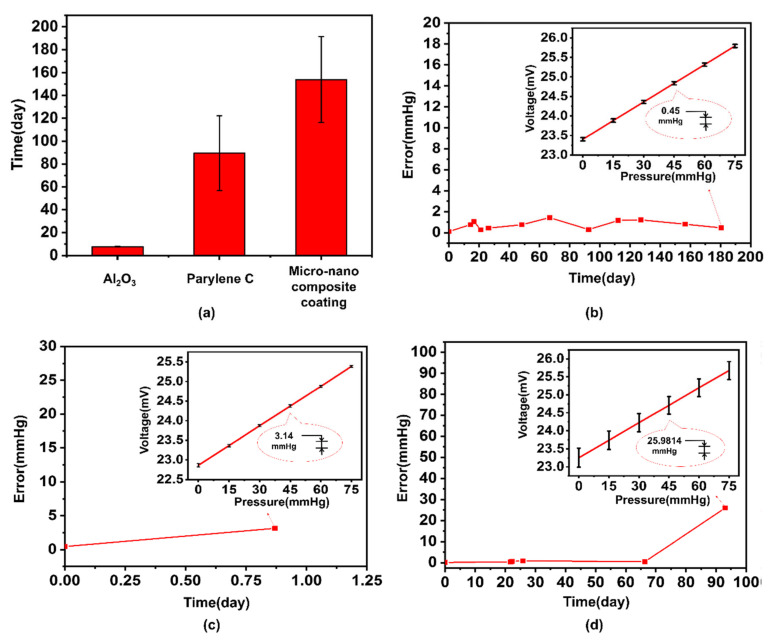
(**a**) Working life of sensors with three encapsulation methods in simulated body fluids; (**b**) relationship between sensor error and waterproof time of the defect filling method of micro-nano composite coating; (**c**) relationship between the error of the sensor coated only with Al_2_O_3_ and the waterproof time; (**d**) relationship between parylene-only sensor error and waterproof time.

We conducted performance durability tests in the simulated human body fluid environment on the devices using three encapsulation methods and regularly measured the error of the device. The test results of the waterproof time are shown in Figure 3a. The average waterproof time of the device encapsulated with nano-scale Al_2_O_3_ was 1.01 days, that of the device encapsulated with Parylene C was 89.5 days, and that of the device encapsulated with the defect filling method of micro-nano composite structure was 153.9 days. After analyzing the waterproof time data obtained by testing the sensors with three different encapsulation structures, we can conclude that the average waterproof days of the device after the micro-nano composite structure encapsulation was greater than the only Al_2_O_3_ or the only Parylene C encapsulation. The sum of the waterproof time of the Parylene C coated device and the Al_2_O_3_ coated device was close to 1.70 times than micro-nano composite coating devices. The phenomenon that the waterproof time is greatly improved shows that the waterproof effect of the double-layer micro-nano composite structure encapsulation is not a simple superposition of the waterproof effects of the two materials, and further mechanism explanations will be given in the discussion section. The simulated body fluid in the experiment was 0.9% normal saline.

### 4.2. Influence of Waterproof Time on Device Error

We separately analyzed the relationship between the error of the device and waterproof time, and characterized the relationship curve in Figure 3b–d. The curves in the figure show that after the durability test, the devices of the three encapsulation methods had different degrees of error changes after soaking for a period of time. The test results in Figure 3b show that the device with the micro-nano composite structure encapsulation remained in good performance after working in simulated body fluid for 180 days. However, the device coated with only Al_2_O_3_ failed after working in simulated body fluid for less than 1 day, as shown in Figure 3c. The device coated with Parylene C only worked properly for 112 days in simulated body fluid, as shown in Figure 3d. Overall, the sensor with double-layer micro-nano composite coating has a long-term stability that is superior to the two other encapsulation methods, whether it is in error variation or waterproof time length. The proposed method can be better used for waterproof encapsulation of the device.

### 4.3. Influence of Encapsulation Method on Device Performance

**Figure 4:**
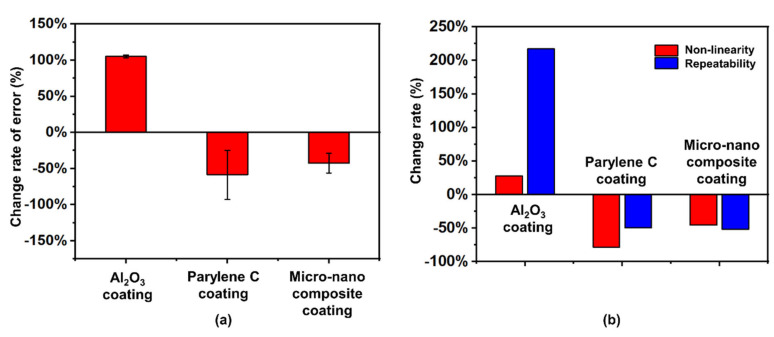
(**a**) Change rate of error of the device before and after encapsulation in different control groups; (**b**) change rate of the device performance parameters before and after different encapsulations.

During the experiment, we found that Al_2_O_3_ is too dense and rigid to coat directly onto the sensitive area of the pressure sensor because it may weaken the elastic deformation of the sensitive element and affect the performances of the sensor (including the non-linear error, repeatability error, hysteresis error, and other indicators), which will reduce the sensitivity of sensor sensitive components and increase the error. Among them, it had the most significant effect on the error. For example, after the device was coated with a single layer of Al_2_O_3_, its error was increased. After the experiment, we found that the error value of the device before encapsulation was 0.2436 mmHg, while it was 0.5003 mmHg after encapsulation; the error change rate reached −105.39%. In terms of device error, the experimental results show that the defect filling method of micro-nano composite structure improved the error to a certain extent, as illustrated in Figure 4a. The single-layer Parylene C and the micro-nano composite structure encapsulated devices had a slight decrease in error, and the rates of error change were 58.95% and 38.46%, respectively. The reason is that the Al_2_O_3_ material in the micro-nano composite structure encapsulation was not directly in contact with the device and it did not negatively affect the error by the Al_2_O_3_ layer.

According to the error theory, we selected several key indicators as examples for analysis. For example, the non-linearity and repeatability of the device after the atomic layer deposition of Al_2_O_3_ dropped substantially: the change rate of repeatability was more than 200%, but the two indicators were improved for the devices over Parylene C encapsulation. Similarly, the device with double-layer micro-nano composite structure encapsulation improved the adverse effects of Al_2_O_3_ encapsulation on devices in nonlinearity and repeatability. The performance change of the device is shown in Figure 4b. The experimental results showed that the double-layer micro-nano composite structure encapsulation highlighted the influence of Parylene C encapsulation on the deformation of sensitive components in the sensor, which can be buffered by the dense, large elastic modulus of Al_2_O_3_. The superiority of this encapsulation method in device encapsulation and the defect filling method of micro-nano composite structure can achieve a better waterproof effect.

The double-layer micro-nano encapsulation structure proposed in this paper can use Parylene C film with good flexibility as a base buffer material between the nanomaterial and the sensor, separating Al_2_O_3_ from the sensitive components, preventing Al_2_O_3_ from directly contacting the sensor sensitive components, and reducing the effect of the dense and large elastic Al_2_O_3_ encapsulation on the movable parts, which was verified from the experimental results. In general, the double-layer micro-nano composite structure encapsulation can reduce the effect of the encapsulation material on the movable parts, which is longer than the sum of the waterproof time of the two single-layer micro-nano encapsulation methods. Moreover, the thickness of this encapsulation method is extremely thin. After encapsulation, the sensitivity of the sensor only decreased by about 1.3%. Therefore, the defect filling method of micro-nano composite structure based on Parylene coating proposed in this paper can achieve the double guarantee of waterproof time and sensor performance.

## 5. Discussion

The experimental results in Figure 3 show that the device with CVD Parylene C and ALD Al_2_O_3_ double-layer encapsulation had a relatively long waterproof time. The waterproof time of the double-layer encapsulation made of two materials was longer than the sum of the waterproof time of the individual encapsulation of each material, and the same material kept the same thickness in the comparative experiment. This finding means that the waterproof effect of micro–nano double-layer encapsulation made of two kinds of materials is not simply the superposition of single-layer effects but also the other increase of waterproof effect. The composite of micro-nano double-layer materials promotes the overall waterproof ability on the original basis.

The proposed defect filling method of micro-nano composite structure can explain and prove this phenomenon well. Pinholes, microcracks, and other defects were observed on the surface of Parylene C, which were directly exposed to water in the case of a single layer. Using the defect filling method, a thin Al_2_O_3_ film will be deposited on Parylene C by atomic layer deposition technology, covering the lower layer compactly, uniformly, and conformally. Under the atomic layer deposition with low-rate deposition conditions, Al_2_O_3_ nanoparticles had sufficient time and space to fill the surface defects of Parylene C material and continue multi-cycle deposition, thus forming a compact 50 nm Al_2_O_3_ layer. From the perspective of the anti-permeability, on the one hand, the overall waterproof ability of the encapsulation increased, which is a simple addition of the waterproof effect of the original Parylene C single layer. On the other hand, the surface defects of Parylene C polymer will be filled with Al_2_O_3_ nanoparticles, and these defects will be filled and separated into many smaller voids on the micro-level. Therefore, the permeation path of water molecules in the Parylene C encapsulation layer was prolonged, and the permeation space was reduced. Thus, the rapid permeation of water molecules through defects is effectively hindered, and the waterproof ability can be additionally improved with Parylene C and ALD Al_2_O_3_ micro-nano composite structure encapsulation. In the experiment, the life days of the double-layer coated device group was not the simple sum of the life days of the single-layer control group. The results of control experiments show that the micro-nano composite coating proposed in this paper achieved good results for the encapsulation of implantable devices.

The defect filling method of micro-nano composite structure proposed in this paper has other advantages for implantable pressure sensors. Theoretically, the waterproof ability of dense ALD Al_2_O_3_ is better than that of polymer Parylene C. However, if ALD Al_2_O_3_ is used alone for encapsulation, the coating thickness and deposit ALD Al_2_O_3_ on the device surface must be increased, which will lead to several serious problems. The Al_2_O_3_ encapsulation layer is dense and has a large elastic modulus. In the experiment of the only ALD Al_2_O_3_ encapsulation, we found that although the Al_2_O_3_ encapsulation layer has only nanometer thickness, if it is directly covered in the sensitive area of the pressure sensor, the elastic deformation of the sensitive element will be weakened, which will lead to the decrease of sensitivity and the increase of error of the sensor sensitive element. However, in the defect filling method of micro-nano composite structure proposed in this paper, Parylene C, a polymer material with good flexibility and low elastic modulus, was used as the inner anti-permeability coating, which has minimal effect on the elastic deformation of sensitive elements. Then, the surface of Parylene C was filled with nano-scale ultra-thin ALD Al_2_O_3_, which compensated for the permeation defects of Parylene C. However, Parylene C flexible material was used as the internal buffer layer of the encapsulation, which avoided the direct contact between Al_2_O_3_ and sensor sensitive elements and reduced the loss of device sensitivity. These two layers were thin. Thus, after double-layer encapsulation, the experiment shows that the sensitivity of the sensor only decreased by about 2%. The experimental results in Figure 4 show that the double-layer encapsulation can also reduce the measurement error of the device to some extent. In summary, the micro-nano composite coating integrates the advantages of two materials, overcomes their respective shortcomings, and realizes the double guarantee of waterproof performance and sensor performance.

Why is the measurement error of the pressure sensor reduced after the defect filling method of micro-nano composite structure encapsulation? Our analysis shows that the micro-nano composite action of the defect filling method reduced the error of the pressure sensor. First, the micro-nano composite structure coating had good barrier protection ability, which can isolate the medium effect on the performance of the pressure sensor in the external environment such as the corrosion of water molecules and oxygen. Second, the Parylene C coating in the micro-nano composite structure had low thermal conductivity, which reduced the interference of the temperature change of the external environment on the pressure sensor. Third, the gap between the Parylene C coating and the movable parts in the sensor was effectively filled because the nano-scale Al_2_O_3_ particles in the micro-nano composite structure were like “rivets”. The overall mechanical properties of the sensor are improved such that the combination between the Parylene C coating and the movable parts was more stable. Fourth, the light and thin micro-nano-composite-coating will not affect the flexibility of the movable structure in the pressure sensor and the output of the sensor signal. Therefore, the encapsulation method of the defect filling method of micro-nano composite coating can reduce nonlinear error and repeatability error of the sensor and make the output of the pressure sensor more accurate.

## 6. Conclusions

In this paper, aimed at solving the water penetration problem caused by the surface and internal defects with parylene fabrication, a defect filling method is proposed based on the diffusion mechanism of water molecules in the polymer membrane. According to the proposed method, a feasible method to prepare such a micro-nano composite coating with parylene is also proposed. This method and its waterproofing effects are studied and verified through its encapsulation on pressure sensors. The experimental results show that the encapsulated sensor has superior long-term stability in vivo, while maintaining high sensitivity and accuracy. The sensitivity of the pressure sensor decreased by only 1.3% after using the proposed micro-nano composite coating encapsulation even though the coated material was attached to the sensitive area of the sensor. The error was reduced by 43% and the devices remained in a good performance after working in the simulated body fluid for 180 days. By contrast, the devices coated with only Al_2_O_3_ deposited by the atomic layer showed a 105% sharp fall in errors and the average waterproof time of the devices was only 1.01 days. The average waterproof time of the devices coated with only Parylene C deposited by the chemical vapor was 112 days. Therefore, the results show that the proposed method is plausible and practicable. The new encapsulation method has the advantages of ultra-thin thickness, high adhesion, strong robustness, and good protection for body fluids, minimal impact on device performance and size, and good biocompatibility. It has a promising application prospects in the field of the industrial devices working in harsh environments and implantable devices such as implantable sensors.

## Figures and Tables

**Table 1:** Comparison of waterproof properties with polymer materials [39].

Polymer	Water Vapor Transmission Rate (g·mm)/(m^2^·day)
Parylene C	0.08
Parylene D	0.09
Parylene HT	0.22
Parylene N	0.59
Polyurethane	0.93–3.4
Epoxy	0.94
Acrylic	13.9

## Data Availability

Data of this research is available upon request via corresponding author.
